# RETRACTION: Rutin, a Flavonoid That Is a Main Component of *Saussurea involucrata*, Attenuates the Senescence Effect in D‐Galactose Aging Mouse Model

**DOI:** 10.1155/ecam/9765215

**Published:** 2026-05-11

**Authors:** Evidence-Based Complementary and Alternative Medicine

RETRACTION: Y.‐C. Yang, H.‐Y. Lin, K.‐Y. Su, et al., “Rutin, a Flavonoid That Is a Main Component of *Saussurea involucrata*, Attenuates the Senescence Effect in D‐Galactose Aging Mouse Model,” *Evidence-Based Complementary and Alternative Medicine*, vol. 2012 (2012). https://doi.org/10.1155/2012/980276.

The above article, published online on 16 July 2012 in Wiley Online Library (wileyonlinelibrary.com), has been retracted by John Wiley & Sons Ltd.

The retraction has been agreed following an investigation of the concerns raised by Actinopolyspora biskrensis [1], which identified several instances of figure duplication as follows:

- Figure 2c: The H&E staining of D‐gal and Rutin+ D‐gal groups appears identical, and the PBS and SI+ D‐gal panels also show unexpected similarity. The latter panel appears in Figure 5d (right) of [2], where it depicts mouse tissue obtained under different experimental conditions.
- Figure 4a (right): The Western Blot bands corresponding to the expression of COX‐2 and iNOS appear identical.
- Figure 4b (right): The bands showing the expression of iNOS show similar features to the COX‐2 bands on the left side of Figure 4a.

The authors provided a response to the above concerns; however, this was not sufficient to guarantee the integrity of the results. The conclusions of this article can no longer be considered reliable, and it is therefore retracted.

The authors have been informed of this retraction but did not provide a response.
